# Resting-state prefrontal EEG biomarkers in correlation with MMSE scores in elderly individuals

**DOI:** 10.1038/s41598-019-46789-2

**Published:** 2019-07-18

**Authors:** Jungmi Choi, Boncho Ku, Young Gooun You, Miok Jo, Minji Kwon, Youyoung Choi, Segyeong Jung, Soyoung Ryu, Eunjeong Park, Hoyeon Go, Gahye Kim, Wonseok Cha, Jaeuk U. Kim

**Affiliations:** 1 Human Anti-Aging Standards Research Institute, Uiryeong-gun, Gyeongsangnam-do Republic of Korea; 2 Korea Institute of Oriental Medicine, Yusung-gu, Deajon, Republic of Korea; 3 Uiryeong Community Health Center, Uiryeong-gun, Gyeongsangnam-do Republic of Korea; 4 Semyung University, Jecheon-si, Chungcheongbuk-do Republic of Korea

**Keywords:** Diagnostic markers, Electroencephalography - EEG, Diagnostic markers

## Abstract

We investigated whether cognitive decline could be explained by resting-state electroencephalography (EEG) biomarkers measured in prefrontal regions that reflect the slowing of intrinsic EEG oscillations. In an aged population dwelling in a rural community (total = 496, males = 165, females = 331), we estimated the global cognitive decline using the Mini-Mental State Examination (MMSE) and measured resting-state EEG parameters at the prefrontal regions of Fp1 and Fp2 in an eyes-closed state. Using a tertile split method, the subjects were classified as T3 (MMSE 28–30, N = 162), T2 (MMSE 25–27, N = 179), or T1 (MMSE ≤ 24, N = 155). The EEG slowing biomarkers of the median frequency, peak frequency and alpha-to-theta ratio decreased as the MMSE scores decreased from T2 to T1 for both sexes (−5.19 ≤ t-value ≤ −3.41 for males and −7.24 ≤ t-value ≤ −4.43 for females) after adjusting for age and education level. Using a double cross-validation procedure, we developed a prediction model for the MMSE scores using the EEG slowing biomarkers and demographic covariates of sex, age and education level. The maximum intraclass correlation coefficient between the MMSE scores and model-predicted values was 0.757 with RMSE = 2.685. The resting-state EEG biomarkers showed significant changes in people with early cognitive decline and correlated well with the MMSE scores. Resting-state EEG slowing measured in the prefrontal regions may be useful for the screening and follow-up of global cognitive decline in elderly individuals.

## Introduction

With the rapid aging of the population, the number of patients with cognitive impairment is quickly increasing^1^; over 46 million people were reported to be living with dementia worldwide in 2015, and this number is estimated to increase to 131.5 million by 2050^2^. Among the many causes of dementia, Alzheimer’s disease (AD) is the most prevalent form in elderly individuals, accounting for approximately 60–80% of cases^3^. The two competing pathologies of AD include the progressive accumulation of beta-amyloid plaques outside neurons and tau tangles inside neurons in the brain. The prevalence of AD doubles for each 5-year increase in age from 65 to 90 years^4^.

At the clinical manifestation stage of dementia, irreversible brain damage is already present. Currently, no cure for AD dementia exists. Therefore, detecting cognitive impairment due to AD at its early stages is urgently needed for therapeutic treatment to slow or halt disease progression. The key to this success is discovering biomarkers that can distinguish mild cognitive impairment (MCI) from normal cognitive aging^5^. The general diagnostic procedures for dementia include clinical interviews with neuropsychological tests, imaging techniques such as structural and functional magnetic resonance imaging (fMRI), and positron emission tomography (PET). MRI is widely available but costly, and it is not suitable for patients who are claustrophobic, while PET scans are expensive, not readily available, and invasive because they involve intravenous access and exposure to radiation^6,7^.

The use of electroencephalography (EEG) for the diagnosis of dementia is a viable option; it is widely available in neurological clinics, inexpensive, noninvasive, and potentially portable^8–12^. EEG has shown potential in identifying the earliest signs of brain dysfunction in subjects with MCI or dementia^13^. Among the diverse approaches of EEG, quantitative analysis of EEG rhythms in subjects who are awake and at rest, which is the simplest method in terms of experimental design, is widely studied and is an easily accessible neurophysiological method for examining dementia^12–22^.

While most previous studies have compared EEG biomarkers in dementia patients, healthy controls and patients with MCI^12,23–25^, the focus of the present study was to observe changes in EEG biomarkers and correlate them with Mini-Mental State Examination (MMSE) scores in an elderly population. The MMSE is used to evaluate the global cognitive status and has been routinely used in clinical settings, particularly for screening for dementia and MCI^26^.

Three major effects of cognitive decline have been observed in the EEG data of AD patients: EEG slowing in terms of a shift in the power spectrum to lower frequencies^27–31^, reduced complexity and reduced coherence^32–38^. Moreover, advances in EEG technology have resulted in single, wireless prefrontal electrode systems with improved usability and portability while maintaining data quality^39,40^. If few-channel EEG systems with dry electrodes that can preferably perform measurements at the prefrontal region with wireless data communication show reliable data quality and significant clinical results, their use will rapidly increase, especially in primary clinics and healthcare-related businesses. The objective of this study was to investigate whether the severity of cognitive decline could be explained by resting, eyes-closed prefrontal EEG biomarkers, with the aim of identifying low-cost, easily accessible, portable, and noninvasive biomarkers that could aid in the early detection of cognitive decline and in the prevention of dementia. To this end, in this study, we tested the hypothesis that three promising biomarkers of resting EEG rhythms are correlated with global cognitive status, as estimated by the MMSE.

## Materials and Methods

### Subjects

From September 2017 to January 2018, a total of 496 elderly participants of age 50 years or older were recruited from 13 public health centers and two long-term care hospitals located in Uiryeong County, Korea. This observational study was conducted as part of the Brain Aging Map Project (BAMP), which is a community welfare project conducted in Uiryeong (a summary of BAMP can be found at http://uiryeong.org, in Korean)^41^. Participants were volunteering County dwellers who could participate approximately 90 minutes of measurement program in which the MMSE, GDS, EEG, PPG and Bio-impedance were included. Individuals who were not in the appropriate condition for the measurement were excluded from the study: who ate food or did intensive physical exercise within an hour of the measurement program, slept less than four hours, had a deformity on electrode contact sites, or who were considered inappropriate by the research nurses. Four clinical research nurses were adequately trained for the measurement of EEG and other devices, which were all portable, and they moved in a team around the public health centers and hospitals by a given program schedule. Participants were recruited through a flyer, brochure, poster advertisement, and phone-call. Written informed consent was obtained from each subject or his/her caregiver prior to study participation. The Institutional Review Board of Semyung University (IRB number: SMU-EX-2017-11-001) approved the study protocol.

After recording basic demographic information, the participants were examined with the MMSE-DS^42^, an extensively used Korean version of the MMSE to assess global cognitive decline, and EEG and other devices were conducted. The MMSE-DS is composed of 19 grouped questions (30 individual questions) and 6 categories: (1) orientation to time (5 points) and place (5 points), (2) attention and calculation (5 points), (3) memory registration (3 points) and recall (3 points), (4) language (6 points), (5) visual construction (1 point), and (6) decision making (2 points). The study was performed in accordance with the principles outlined in the Declaration of Helsinki. Our data are available upon request for the reproduction of the results presented in this study.

### EEG recordings

All subjects underwent EEG recordings for five minutes in an upright seated position in a resting state with their eyes closed. A series of other tests were also conducted. These additional tests were beyond the scope of the present study and thus were not reported here. Noninvasive monopolar scalp electrodes recorded the electrical brain activity of the prefrontal regions (Fp1 and Fp2 in the International 10/20 electrode system) with a reference on the right earlobe. The band-pass frequency of the neuroNicle amplifier (LAXTHA Inc., Korea) was 3 to 43 Hz, and the input range was +/−393 µV (Input noise <0.6 µVrms); All filters were digital, and IIR Butterworth filters were applied. Band-stop: 2nd order with f1 = 55 Hz and f2 = 65 Hz. High-pass filter: 1st order with fc = 2.6 Hz. Low-pass filter: 8th order with fc = 43 Hz^43^. All contact impedances were kept below 10 kΩ. All data were digitized in continuous recording mode (5 minutes of EEG; 250 Hz sampling rate; 15-bit resolution).

To minimize ocular, muscular, and other types of artifacts, a trained operator monitored the subject and EEG traces, instructed the subject to remain in a state with their eyes closed and muscles relaxed in a quiet environment and alerted the subject whenever he/she showed signs of behavioral or EEG drowsiness. None of the resting-state, eyes-closed EEG data were rejected due to artifacts in this study.

### EEG biomarker and computation

The frequency-domain (or spectral-domain) features are typically used in the quantitative analysis of EEG rhythms. To transform the EEG signal from the time domain to the frequency domain, a Fourier transform of the autocorrelation function was employed to provide the power spectral density.

In resting-state eyes-closed EEG, intrinsic oscillations reflecting an idling cortical state become dominant, and the dominant peak frequency usually occurs in the 5–12 Hz band. Previous reports have commonly shown that the dominant oscillatory frequencies that appear in the alpha band during normal aging become lower in patients with cognitive disorder^12,13,25^.

The present study focused on the following EEG biomarkers (EEG variables) to explain the slowing of brain rhythms in a resting eyes-closed state: median frequency (MDF), peak frequency (PF) and the alpha-to-theta ratio (ATR), which have been reported to be suitable classification biomarkers for Alzheimer’s disease and mild cognitive impairment^17,18^. These biomarkers were derived from a frequency-domain analysis of EEG data measured for 5 minutes; the MDF measures the median frequency, and the PF measures the frequency at the maximum peak in the dominant intrinsic oscillatory frequency band of 4–13 Hz of the EEG power spectrum. The ATR measures the power ratio of alpha rhythms (8–13 Hz) to theta rhythms (4–8 Hz). The variables were averaged over the left and right EEG signals. Both of the MDF and PF are in units of Hz and the ATR is in unitless; the MDF ranged between 4.31 and 10.80 Hz, the PF ranged between 5.71 and 12.89 Hz, and the ATR ranged between 0.43 and 2.72 for the study participants.

The EEG power spectrum was obtained by fast Fourier transform (FFT) of the EEG signals within a rectangular window. The MDF was calculated in two steps: (1) All the spectral power values in the 4–13 Hz frequency domain were summed and divided by two. (2) The frequency at which the cumulative power in the 4–13 Hz first exceeded the value calculated in step (1) was selected. The PF was determined by the frequency at which the power of the EEG spectrum was the largest in the 4–13 Hz frequency domain. To obtain the ATR, the alpha power and theta power were calculated as follows. (1) Alpha power: The spectral power values in the frequency range from 8 to 13 Hz were summed and converted to the natural logarithmic scale. (2) Theta power: The spectral power values in the frequency range from 4 to 8 Hz were summed and converted to the natural logarithmic scale.

### Reliability of prefrontal EEG biomarkers

To examine the quality of resting-state EEG slowing measured in the prefrontal region, we conducted two tests. First, we compared the MDF, PF and ATR values between the prefrontal and occipital regions using a multichannel EEG device prior to the clinical study (between Fp1 and O1 and between Fp2 and O2) for 31 subjects. We calculated the intra-class correlation coefficients (ICC) and mean differences of the MDF, PF and ATR between Fp1 and O1 and between Fp2 and O2 (Table A1 in the appendix). Between Fp1 and O1, the ICCs were 0.865, 0.936 and 0.541 for the MDF, PF and ATR, respectively. Between Fp2 and O2, the ICCs were 0.690, 0.951 and 0.440 for the MDF, PF and ATR, respectively. Details were shown in Table S1 in Supplementary Materials. These findings indicate that the variables related to the alpha peak, such as MDF and PF, contain the same clinical information and are interchangeable between the prefrontal and occipital regions. However, the ATR, which reflects the relative power between the alpha and theta bands, behaved slightly differently between the two regions, which implies that the clinical interpretations of power-related quantities may not be interchangeable. Despite this difference in ICC for the ATR, we did not observe significant differences in the mean values in any of the variables between the prefrontal and occipital regions. To maintain the same data processing protocol with the main results of this manuscript, none of the resting-state eyes-closed EEG data were rejected for artifacts.

Next, we tested for the data contamination due to muscle and eye movement of the (Fp1, Fp2)-prefrontal EEG signals as we did not reject any artifact in the signal processing. First, we checked that none of the EEG data of all of the participants was contaminated by a large amount of artifacts. Specifically, none of 496 participants contained more than 10% of EEG amplitude exceeding 200 μV; this value is a common exclusion threshold for serious artifacts^44^. When applying more strict voltage threshold, we found still none with 10% of amplitude exceeded 150 μV, and only 2 samples in Fp1 and 1 sample in Fp2 with 100 μV as a threshold.

Second, we randomly selected 45 samples from the entire data set of 496 participants. From each EEG signal, we selected a data slice of one-minute interval where the EEG amplitude confined within ±80 μV, which was a strict condition for least contamination from muscle and eye movements compared to a common threshold of ±200 μV^44^, and calculated the MDF, PF and ATR. Finally, we compared the resulting three variables obtained from the entire EEG recording of 5 minutes and the artifact-free slice. As a result, the mean ± standard deviation was 8.53 ± 0.87 for MDF, 8.69 ± 1.32 for PF and 1.26 ± 0.32 for ATR with full-length EEG recordings, and 8.56 ± 0.85 for MDF, 8.45 ± 1.46 for PF and 1.28 ± 0.34 for ATR with the artifact-free slices. The mean ± standard deviation of differences was 0.03 ± 0.47 for MDF, 0.24 ± 1.26 for PF and 0.03 ± 0.14 for ATR, and the paired t-test showed no statistical difference (p > 0.2) in each variable. Therefore, we concluded that the three EEG variables of MDF, PF and ATR behaved rather stationary during the 5 minutes of EEG recording and the interference due to artifacts could be ignored.

### Statistical analysis

#### Overview

The significance level was set to *α* = 0.05 for all statistical tests. Statistical analysis was conducted using the R statistical software (version 3.4.4, released at 2018-03-15)^45^. The demographics of the participants in Uiryeong County were summarized as the means and standard deviations for continuous variables or as the frequencies and proportions for categorical variables, in accordance with the MMSE cognitive stages. The cognitive stages are divided by the MMSE tertiles (T3: 28 ≤ MMSE ≤ 30; T2: 25 ≤ MMSE ≤ 27; T1: MMSE ≤ 24)^46–49^ to obtain the balanced subgroups (Table 1).

#### Association between EEG variables and MMSE tertiles

**Figure 1:**
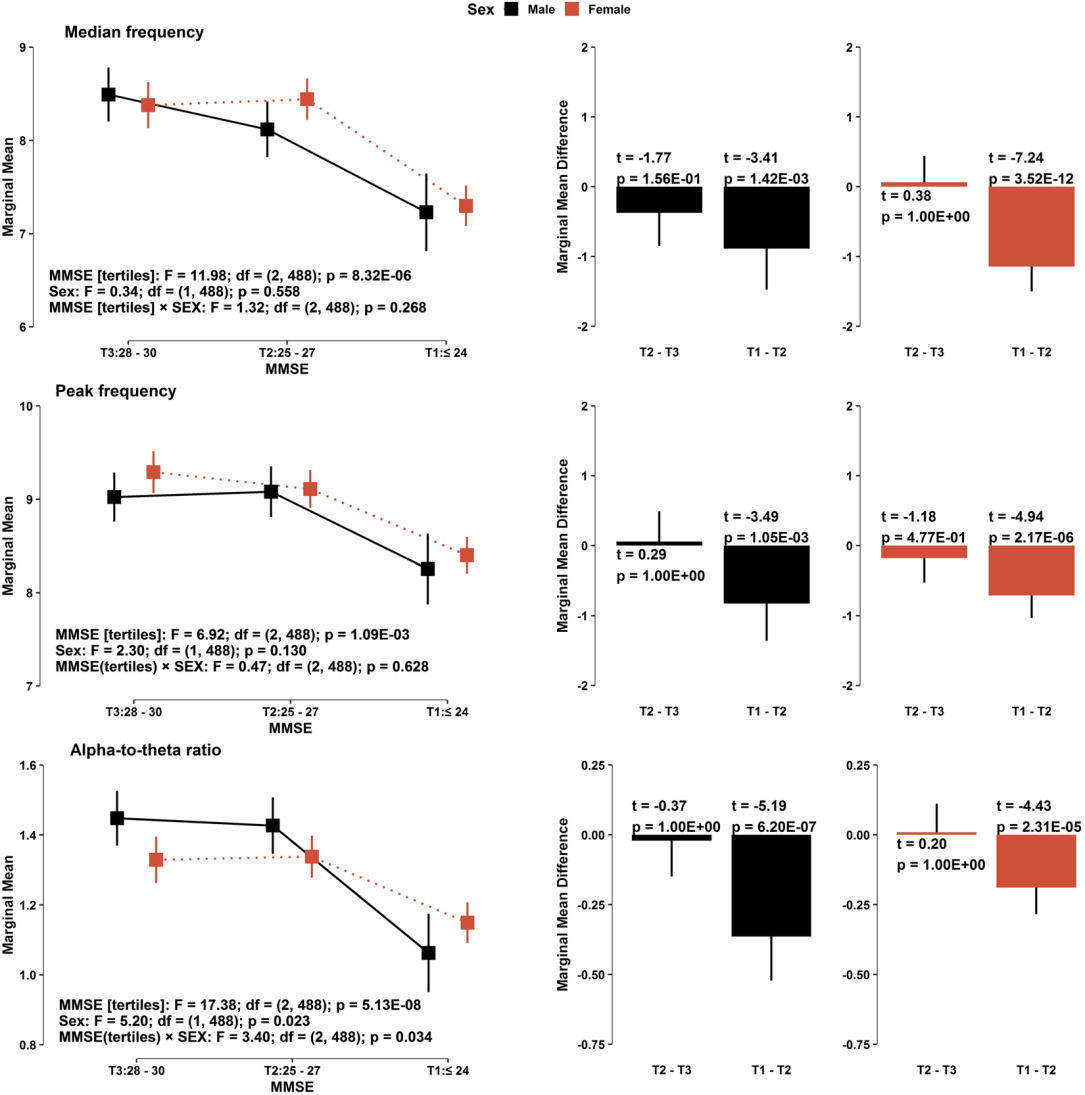
(Left panels) Estimated marginal means of each EEG variable according to sex and (right panels) its consecutive contrasts in the sequence of the MMSE cognitive stages, based on a GLM with the identity function and normal distribution. Each model was adjusted for age and education level and contains the interaction term between sex and cognitive stage. Lines across the squares represent the 95% CIs for the marginal means. Statistics (obtained from the ANOVA table of each GLM: test statistics, degrees of freedom, and p-values) related to the effects of MMSE tertiles, sex, and the interaction term are presented at the left-bottom of each panel on the left. At the top-left of each panel on the right, test statistics (t-values) and p-values related to consecutive contrasts of the MMSE cognitive stage are indicated (with 488 degrees of freedom). P-values and simultaneous 95% CIs for the differences between sequential cognitive stages were corrected by Bonferroni’s adjustment (right panels).

To investigate the association between the cognitive stages of the MMSE and each EEG variable, namely, the MDF, PF, and ATR, a generalized linear model (GLM) with the identity function and normal distribution was used to estimate marginal means in accordance with sex and the level of cognitive decline by including the interaction term between sex and cognitive status and other covariates (age and education level). The analysis of variance test for each GLM for the corresponding EEG variable was conducted to examine the general effects of terms included in the model. The consecutive contrasts for the MMSE tertiles (T2 vs. T3 and T1 vs. T2) were tested by t-statistics. The p-values related to multiple tests for the consecutive MMSE tertiles were adjusted by Bonferroni correction. Similarly, Bonferroni’s 95% simultaneous confidence intervals (CI) were also obtained for each contrast with respect to the GLMs (Fig. 1).

#### Correlation/association analysis between EEG variables and MMSE total and domain scores

**Figure 2:**
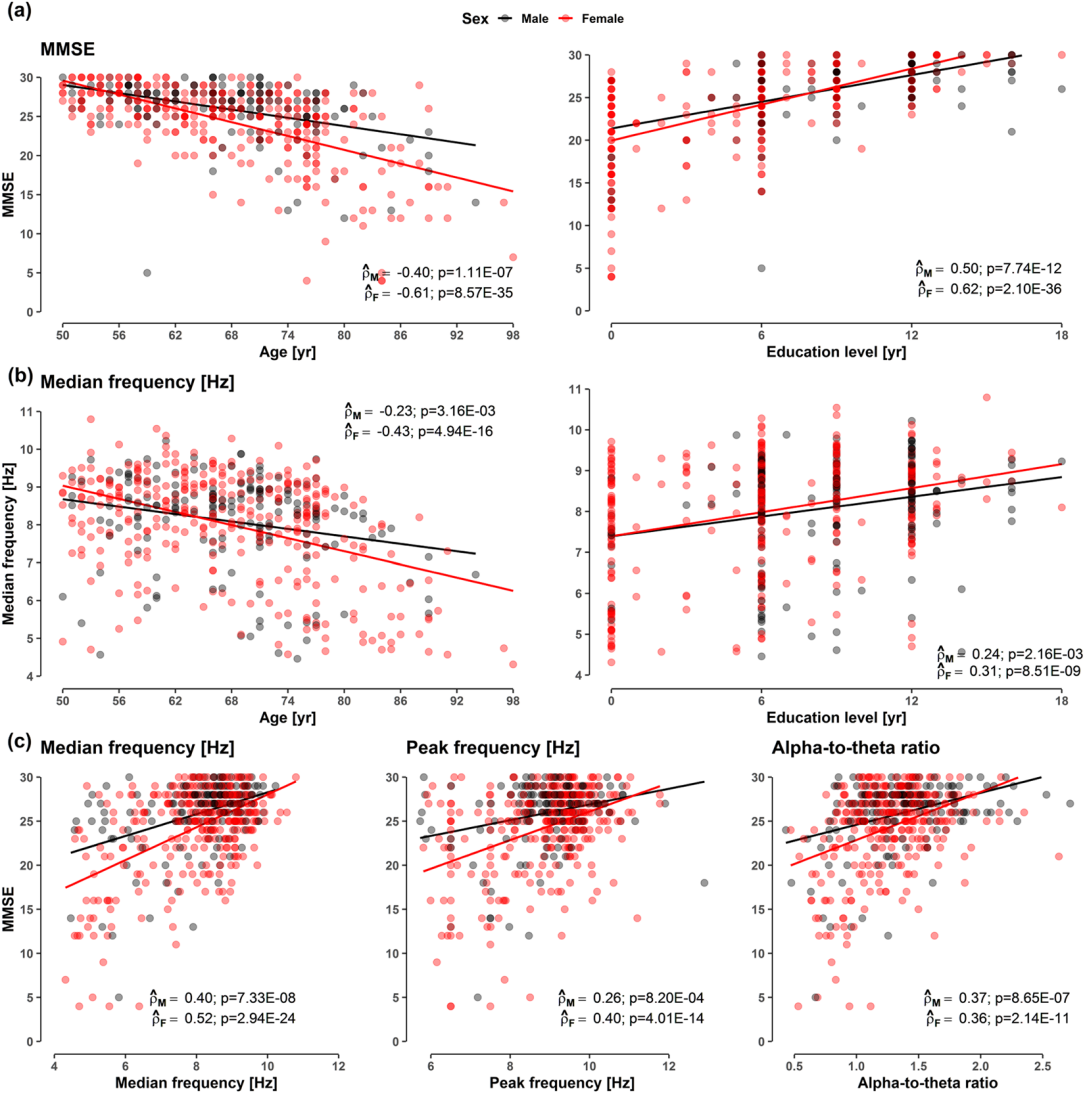
Scatterplots (**a**) between MMSE and demographic variables (age and education levels), (**b**) between MDF and demographic variables, and (**c**) between the MMSE scores and EEG variables (MDF, PF, ATR), according to sex. The simple linear regression curves for MMSE and each EEG variable are denoted on figures according to sex. Pearson correlation coefficients and p-values are noted on each panel according to sex. The rest of the scatterplots are provided in Fig. S2 in Supplementary Materials.

Pearson correlation coefficients (PCCs) were obtained to examine the relationship between the EEG variables and other measures including demographics and the MMSE scores. Estimated correlation coefficients and their corresponding 95% CIs and p-values were summarized in tables (Tables 2–5) and in Fig. 2. The statistical difference between any two correlation coefficients was investigated by several statistical tests provided in the cocor package^50^. Conventional Fisher’s Z transformation to correlation coefficients was employed to perform the Z-test for the comparisons from two independent groups. Zou’s 95% CIs^51^ for the difference of two independent correlation coefficients were obtained. To test two dependent correlation coefficients overlapped with common covariates (e.g. age and education level), Meng’s Z-tests and 95% CIs were employed^52^.

**Figure 3:**
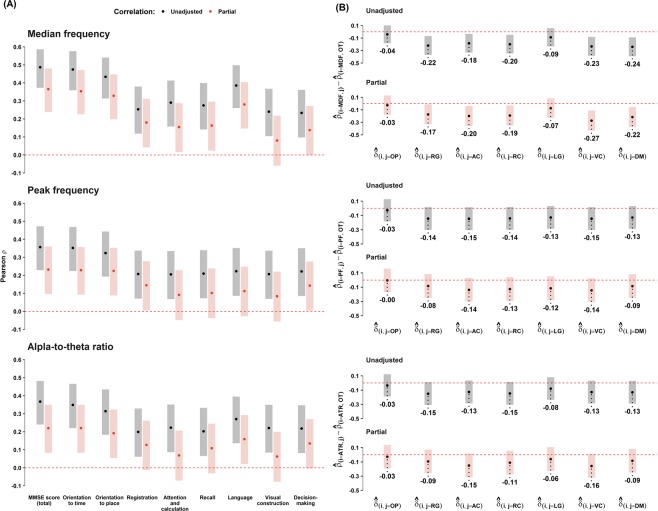
Unadjusted and partial correlation coefficients between EEG variables and MMSE total and subdomain scores. (**A**) Unadjusted (black circles) and partial (red circles) PCCs between EEG variables and MMSE total and domain scores. Partial PCCs are controlled for sex, age, and education level. The lengths of each shaded area represent Bonferroni corrected 95% confidence intervals. The statistical significance for each coefficient can be determined whether the shaded area crosses zero (the red dashed line). (**B**) Difference of pairs of Pearson correlation coefficients between ${\hat{{\boldsymbol{\rho }}}}_{{\boldsymbol{i}},{\boldsymbol{j}}}$ and ${\hat{{\boldsymbol{\rho }}}}_{{\boldsymbol{i}},{\boldsymbol{OT}}}$, where i = {MDF, PF, ATR} and j = {OP, RG, AC, RC, LG, VC, DM}. The x-axis label, ${\hat{{\boldsymbol{\delta }}}}_{({\boldsymbol{i}},{\boldsymbol{j}})}={\hat{{\boldsymbol{\rho }}}}_{({\boldsymbol{i}},{\boldsymbol{j}})}-{\hat{{\boldsymbol{\rho }}}}_{({\boldsymbol{i}},{\boldsymbol{OT}})}$, with the given EEG variables for i = MDF (top panel), i = PF (middle panel), and i = ATR (bottom panel). Statistical tests for the difference between two dependent correlation coefficients are performed by the Z test suggested by Meng *et al*.^52^. Meng’s 95% confidence intervals^52^ for the two correlation difference are represented with shaded bars with different colors: black for unadjusted PCCs and red for partial PCCs. Values below the lower limit of each 95% confidence interval indicates ${\hat{{\boldsymbol{\delta }}}}_{({\boldsymbol{i}},{\boldsymbol{j}})}$ for ***i*** and ***j***. Abbreviation: OT, orientation to time; OP, orientation to place; RG, registration; AC, attention and calculation; RC, recall; LG, language; VC, visual construction; DM, decision making.

Partial PCCs, controlled for sex, age, and education level, were also obtained to examine the relationship among the EEG variables, MMSE total and domain scores. The statistical difference between two partial PCCs for each EEG variable and the MMSE domain scores was also investigated by employing the identical statistical tests used in the correlation analyses described above (Fig. 3). Similarly, we performed multiple linear regression (MLR) analyses to assess the association between each of the EEG variables and the MMSE total and domain scores adjusted for sex, age, and education level. For the relative comparisons among regression coefficients for each EEG variable, standardized regression coefficients were also included in MLR analyses (Table 6).

#### Development of the predictive models for the MMSE total score

Finally, we developed a predictive model for the MMSE total score. For the preprocessing, all continuous predictors were standardized with 0 for the mean and 1 for the standard deviation. The ordinary weighted least squares (WLS) method was used to estimate the MMSE total score. Stepwise variable selection through both forward and backward searches based on Akaike information criterion^53^ was applied to the ordinary WLS model to reduce the model complexity. In addition, we applied a penalized regression model related to the L1 norm penalty, such as the least absolute shrinkage selection operator (LASSO)^54^ and elastic net^55^ methods. Another penalized regression approach, ridge regression^56,57^ with L2 norm penalty was additionally used as a candidate model to address multicollinearity of EEG and demographic varibles. All penalized regression approaches were conducted using the glmnet package^58^ with changing mixing parameter of L1 and L2 norm penalty *α* (ridge regression: *α* = 0; elastic net: 0 < *α* < 1; LASSO: *α* = 1). Two major hyperparameters for penalized regressions in this study were initialized as follows: the regularization parameter *λ* was set to 300 values ranged from exp[log(10)] to exp[log(0.0001)], and the mixing parameter *α* has a value between 0 and 1 in 0.1 intervals (a total of 11). Each candidate model contains identical components: three EEG variables and demographics including sex, age, and education level. Quadratic terms of continuous predictors and the second order interaction terms corresponding to sex (1 for female and 0 for male) were also included in each model.

The total dataset of 496 samples was randomly divided into 80% of the training set (n = 396) and 20% of the test set (n = 100) for the model validation. The training (including nested training and validation sets in each fold of cross-validation) and test sets had approximately identical distributions to the total dataset in the result of stratification by the MMSE tertiles. The test set was not included in the validation procedure; it was only used for the final assessment of the predictive models. For the 10-fold cross-validation, similarly, the training set was divided into 90% (356 to 357) for model development and 10% (39 to 40) for the valiation in each fold. The root mean square error (RMSE) was used to evaluate the performance of the validation set for candidate models including the ordinary WLS and penalized regression models. For each penalized regression model, hyperparamters *λ* and *α* with the minimum RMSE were selected through the 10-fold cross-validation. Among the four candidate models (ordinary WLS, ridge, elastic net, and LASSO), the model with the smallest value of RMSE was finally selected as the predictive model for the MMSE total score. Although only one model was selected as a result of validation, the final four models were developed based on the common training set and were applied to the predicted MMSE scores for the test set, for the purpose of the overall comparison (Tables 6 and 7).

To assess the agreement between the true MMSE total score and predicted MMSE total score in the test set, several statistics were provided in the results. PCCs between the predicted and true MMSE scores were calculated according to each candidate model. The mean difference between those values was also obtained to verify the concordance between the true and predicted MMSE scores. Additionally, the ICC was calculated to determine the reliability of the predicted models. A Bland-Altman plot^59^ for the final model was employed to investigate the limit of agreement (LOA) between the true and predicted MMSE values.

## Results

This section is organized as follows. In Section 3.1, we examined the demographic (sex, age, education level) and neuropsychological characteristics (MMSE total and domain scores) of the participants. Sequentially in Section 3.2, we tested for the association between each EEG variable and the cognitive stage of the MMSE tertiles for each sex. Next, we investigated in-depth the correlations between the MMSE (total) score, EEG variables and demographic variables such as age and education level in Section 3.3, and investigated the association of EEG variables with the MMSE cognitive domains in Section 3.4. Finally, in Section 3.5, we suggested predictive models for the MMSE total score using the three resting-state biomarkers of EEG slowing.

**Table 1:** Demographic characteristics.

	Total	MMSE tertiles		
		T3: 28–30	T2: 25–27	T1: ≤24
N (%)	496 (100.0%)	162 (32.7%)	179 (36.1%)	155 (31.2%)
Sex				
Male	165 (33.27%)	68 (41.98%)	64 (35.75%)	33 (21.29%)
Female	331 (66.73%)	94 (58.02%)	115 (64.25%)	122 (78.71%)
Age [yr]	67.84 ± 9.77 [50.00, 98.00]	63.05 ± 8.37 [50.00, 88.00]	66.54 ± 8.24 [50.00, 89.00]	74.34 ± 9.31 [53.00, 98.00]
Education level [yr]	7.03 ± 4.33 [0.00, 18.00]	9.81 ± 3.35 [0.00, 18.00]	7.45 ± 3.63 [0.00, 18.00]	3.65 ± 3.66 [0.00, 16.00]
MMSE score (total)	24.85 ± 4.69 [4.00, 30.00]	28.73 ± 0.80 [28.00, 30.00]	26.03 ± 0.82 [25.00, 27.00]	19.43 ± 4.71 [4.00, 24.00]
MMSE domains				
Orientation to time	4.38 ± 1.22 [0.00, 5.00]	4.94 ± 0.23 [4.00, 5.00]	4.78 ± 0.49 [3.00, 5.00]	3.34 ± 1.68 [0.00, 5.00]
Orientation to place	4.71 ± 0.83 [0.00, 5.00]	4.99 ± 0.11 [4.00, 5.00]	4.96 ± 0.19 [4.00, 5.00]	4.13 ± 1.28 [0.00, 5.00]
Registration	2.89 ± 0.35 [0.00, 3.00]	2.99 ± 0.11 [2.00, 3.00]	2.97 ± 0.17 [2.00, 3.00]	2.70 ± 0.55 [0.00, 3.00]
Attention and calculation	2.88 ± 1.71 [0.00, 5.00]	4.44 ± 0.70 [3.00, 5.00]	3.03 ± 1.18 [0.00, 5.00]	1.07 ± 1.18 [0.00, 5.00]
Recall	1.88 ± 0.99 [0.00, 3.00]	2.55 ± 0.57 [1.00, 3.00]	1.92 ± 0.81 [0.00, 3.00]	1.12 ± 1.01 [0.00, 3.00]
Language	5.61 ± 0.76 [1.00, 6.00]	5.92 ± 0.27 [5.00, 6.00]	5.80 ± 0.42 [4.00, 6.00]	5.06 ± 1.06 [1.00, 6.00]
Visual construction	0.60 ± 0.49 [0.00, 1.00]	0.90 ± 0.30 [0.00, 1.00]	0.60 ± 0.49 [0.00, 1.00]	0.27 ± 0.45 [0.00, 1.00]
Decision making	1.91 ± 0.33 [0.00, 2.00]	2.00 ± 0.00 [2.00, 2.00]	1.97 ± 0.18 [1.00, 2.00]	1.74 ± 0.51 [0.00, 2.00]

Variables are summarized as the mean ± SD and range [min, max] values in accordance with the MMSE cognitive stages of T3 to T1. The MMSE was further divided into 8 cognitive domains for a detailed score distribution for each cognitive stage from T3 to T1.

### Demographics and neuropsychological characteristics

The overall demographic information and neuropsychological characteristics of the 496 subjects are summarized in Table 1. The percentages of female/male participants were 66.73%/33.27%, and the mean age ± SD was 67.84 ± 9.77 years. According to the severity of cognitive decline, as estimated by the MMSE (MMSE-DS) scores with tertile splitting, we classified subjects into the three cognitive stages: T3 (N = 162), T2 (N = 179) and T1 (N = 155). This classification showed that as age increased and education level decreased, the cognitive status declined. For a detailed analysis, we categorized the MMSE into 8 cognitive domains, namely, orientation to time, orientation to place, memory registration, attention and calculation, memory recall, language, visual construction, and decision making^42^. As shown in Table 1, the scores in each cognitive domain monotonically declined as the MMSE cognitive scores declined from T3 to T1.

**Table 2:** Pearson correlation coefficients between the MMSE, EEG and demographic variables.

	Age				Education level			
	${\hat{{\boldsymbol{\rho }}}}_{{\boldsymbol{T}}}$	${\hat{{\boldsymbol{\rho }}}}_{{\boldsymbol{M}}}$	${\hat{{\boldsymbol{\rho }}}}_{{\boldsymbol{F}}}$	${\hat{{\boldsymbol{\rho }}}}_{{\boldsymbol{F}}}-\,{\hat{{\boldsymbol{\rho }}}}_{{\boldsymbol{M}}}$	${\hat{{\boldsymbol{\rho }}}}_{{\boldsymbol{T}}}$	${\hat{{\boldsymbol{\rho }}}}_{{\boldsymbol{M}}}$	${\hat{{\boldsymbol{\rho }}}}_{{\boldsymbol{F}}}$	${\hat{{\boldsymbol{\rho }}}}_{{\boldsymbol{F}}}-\,{\hat{{\boldsymbol{\rho }}}}_{{\boldsymbol{M}}}$
MMSE	−0.55 (−0.63, −0.46) 2.26E-39	−0.40 (−0.56, −0.20) 8.91E-07	−0.61 (−0.69, −0.50) 6.86E-34	−0.21 (−0.42, −0.01) 2.58E-02	0.60 (0.52, 0.68) 1.24E-49	0.50 (0.32, 0.64) 6.20E-11	0.62 (0.52, 0.70) 1.68E-35	0.12 (−0.06, 0.32) 5.64E-01
Median frequency	−0.37 (−0.47, −0.26) 1.10E-16	−0.23 (−0.42, −0.02) 2.53E-02	−0.43 (−0.54, −0.30) 3.95E-15	−0.20 (−0.44, 0.03) 1.64E-01	0.29 (0.17, 0.40) 5.67E-10	0.24 (0.03, 0.43) 1.73E-02	0.31 (0.17, 0.44) 6.81E-08	0.07 (−0.16, 0.32) 1.00E + 00
Peak frequency	−0.34 (−0.45, −0.23) 3.54E-14	−0.19 (−0.38, 0.03) 1.27E-01	−0.40 (−0.52, −0.27) 1.89E-13	−0.22 (−0.46, 0.02) 1.07E-01	0.22 (0.10, 0.33) 7.99E-06	0.16 (−0.06, 0.36) 3.71E-01	0.25 (0.11, 0.39) 2.15E-05	0.10 (−0.15, 0.35) 1.00E + 00
Alpha-to-theta ratio	−0.30 (−0.41, −0.18) 9.07E-11	−0.26 (−0.44, −0.05) 7.28E-03	−0.33 (−0.46, −0.19) 5.67E-09	−0.07 (−0.32, 0.16) 1.00E + 00	0.28 (0.16, 0.39) 1.63E-09	0.37 (0.17, 0.54) 9.52E-06	0.21 (0.06, 0.35) 1.26E-03	−0.16 (−0.39, 0.08) 5.30E-01

${\hat{\rho }}_{T},\,{\hat{\rho }}_{M},{\rm{a}}{\rm{n}}{\rm{d}}\,{\hat{\rho }}_{F}$ are the Pearson correlation coefficients for the total, male and female group, respectively. The difference of two independent correlation coefficients between female and male groups (${\hat{\rho }}_{F}\,-{\hat{\rho }}_{M}$) is tested by Fisher’s Z test. Zou’s 95% confidence intervals for correlation differences between sexes are noted inside of parentheses.

Each cell contains the estimated correlation coefficient or the difference between two correlation coefficients according to the column label (the first row), 95% confidence interval (the second row), and p-value (the third row). Obtained p-values and 95% CIs are corrected by Bonferroni adjustment.

**Table 3:** The difference between pairs of Pearson correlation coefficients in Table 2.

	Total		Male		Female	
	${\hat{{\boldsymbol{\rho }}}}_{{\boldsymbol{i}},{\bf{a}}{\bf{g}}{\bf{e}}}-\,{\hat{{\boldsymbol{\rho }}}}_{{\bf{M}}{\bf{M}}{\bf{S}}{\bf{E}},{\bf{a}}{\bf{g}}{\bf{e}}}$	${\hat{{\boldsymbol{\rho }}}}_{{\boldsymbol{i}},{\boldsymbol{edu}}}-\,{\hat{{\boldsymbol{\rho }}}}_{{\bf{M}}{\bf{M}}{\bf{S}}{\bf{E}},{\bf{e}}{\bf{d}}{\bf{u}}}$	${\hat{{\boldsymbol{\rho }}}}_{{\boldsymbol{i}},{\boldsymbol{age}}}-\,{\hat{{\boldsymbol{\rho }}}}_{{\bf{M}}{\bf{M}}{\bf{S}}{\bf{E}},{\bf{a}}{\bf{g}}{\bf{e}}}$	${\hat{{\boldsymbol{\rho }}}}_{{\boldsymbol{i}},{\bf{e}}{\bf{d}}{\bf{u}}}-\,{\hat{{\boldsymbol{\rho }}}}_{{\bf{M}}{\bf{M}}{\bf{S}}{\bf{E}},{\bf{e}}{\bf{d}}{\bf{u}}}$	${\hat{{\boldsymbol{\rho }}}}_{{\boldsymbol{i}},{\bf{a}}{\bf{g}}{\bf{e}}}-\,{\hat{{\boldsymbol{\rho }}}}_{{\bf{M}}{\bf{M}}{\bf{S}}{\bf{E}},{\bf{a}}{\bf{g}}{\bf{e}}}$	${\hat{{\boldsymbol{\rho }}}}_{{\boldsymbol{i}},{\bf{e}}{\bf{d}}{\bf{u}}}-\,{\hat{{\boldsymbol{\rho }}}}_{{\bf{M}}{\bf{M}}{\bf{S}}{\bf{E}},{\bf{e}}{\bf{d}}{\bf{u}}}$
i = MDF	0.18 (0.03, 0.43) 1.23E-02	−0.32 (−0.60, −0.20) 3.00E-08	0.17 (−0.15, 0.53) 1.00E + 00	−0.26 (−0.65, 0.04) 1.34E-01	0.18 (0.00, 0.50) 4.41E-02	−0.31 (−0.65, −0.16) 1.68E-05
i = PF	0.21 (0.06, 0.46) 1.83E-03	−0.39 (−0.68, −0.28) 1.43E-11	0.21 (−0.11, 0.57) 7.34E-01	−0.35 (−0.74, −0.05) 1.10E-02	0.20 (0.03, 0.52) 1.35E-02	−0.36 (−0.71, −0.22) 2.93E-07
i = ATR	0.25 (0.11, 0.51) 6.63E-05	−0.32 (−0.61, −0.21) 1.49E-08	0.14 (−0.18, 0.50) 1.00E + 00	−0.13 (−0.51, 0.18) 1.00E + 00	0.28 (0.12, 0.61) 1.93E-04	−0.41 (−0.76, −0.27) 5.99E-09

Difference between a pair of Pearson correlation coefficients ${\hat{\rho }}_{i,j}$ and ${\hat{\rho }}_{{\rm{MMSE}},j}$ for the total, male and female group, where i = {MDF, PF, ATR} and j = {age, edu}. The statistical test was performed based on the Z test suggested by Meng *et al*.^52^, for testing the difference between two dependent correlation coefficients overlapped with common demographic variables. Meng’s 95% confidence intervals^52^ for the differences are noted inside of parentheses.

Each cell contains the difference between two correlation coefficients according to the column label (the first row), 95% confidence interval (the second row), and p-value (the third row). Obtained p-values and 95% CIs are corrected by Bonferroni adjustment.

### Association of resting-state EEG slowing according to the MMSE cognitive stages

We tested for the association between each EEG variable and the cognitive stages of the MMSE tertiles for each sex using a generalized linear model, which was adjusted for age and education level and contains the interaction term between sex and cognitive stage (Fig. 1). As shown in the left panels of Fig. 1, the marginal mean of each EEG variable decreased monotonically as the MMSE cognitive stages declined from T3 to T1, and there was no interaction between sex and cognitive status in the MDF and PF, while the interaction was marginal in the ATR. In the analysis of the difference of the marginal means between adjacent cognitive stages, significant differences remained between T1 and T2 for both male and female subjects (t-value equals to −3.41, −3.49, −5.19 for males and −7.24, −4.94 and −4.43 for females in the MDF, PF and ATR, respectively), while no such differences were found between T2 and T3 (the right panels of Fig. 1). The ATR showed relatively strong difference between T1 and T2 (t = −5.19) for males, reflecting the marginal interaction between sex and cognitive status. The strongest association was shown in females between the MDF and the MMSE cognitive score.

**Table 4:** Pearson correlation coefficients between the MMSE score and EEG variables.

	MMSE			
	${\hat{{\boldsymbol{\rho }}}}_{{\boldsymbol{T}}}$	${\hat{{\boldsymbol{\rho }}}}_{{\boldsymbol{M}}}$	${\hat{{\boldsymbol{\rho }}}}_{{\boldsymbol{F}}}$	${\hat{{\boldsymbol{\rho }}}}_{{\boldsymbol{F}}}-\,{\hat{{\boldsymbol{\rho }}}}_{{\boldsymbol{M}}}$
Median frequency	0.49 (0.40, 0.56) 2.16E-30	0.40 (0.24, 0.55) 2.20E-07	0.52 (0.42, 0.61) 8.82E-24	−0.01 (−0.21, 0.19) 1.00E + 00
Peak frequency	0.36 (0.26, 0.45) 7.70E-16	0.26 (0.08, 0.42) 2.46E-03	0.40 (0.28, 0.50) 1.20E-13	0.12 (−0.06, 0.31) 3.78E-01
Alpha-to-theta ratio	0.37 (0.27, 0.46) 8.28E-17	0.37 (0.20, 0.52) 2.60E-06	0.36 (0.24, 0.47) 6.43E-11	0.14 (−0.06, 0.35) 2.92E-01

The details are identical with Table 2.

**Table 5:** Differences of pairs of Pearson correlation coefficients in Table 4.

	Total		Male		Female	
	${\hat{{\boldsymbol{\rho }}}}_{{\boldsymbol{i}},{\bf{\text{MMSE}}}}{\boldsymbol{-}}\,{\hat{{\boldsymbol{\rho }}}}_{{\boldsymbol{PF}}{\boldsymbol{,}}{\bf{\text{MMSE}}}}$	${\hat{{\boldsymbol{\rho }}}}_{{\boldsymbol{i}}{\boldsymbol{,}}{\bf{\text{MMSE}}}}{\boldsymbol{-}}\,{\hat{{\boldsymbol{\rho }}}}_{{\boldsymbol{ATR}}{\boldsymbol{,}}{\bf{\text{MMSE}}}}$	${\hat{{\boldsymbol{\rho }}}}_{{\boldsymbol{i}}{\boldsymbol{,}}{\bf{\text{MMSE}}}}{\boldsymbol{-}}\,{\hat{{\boldsymbol{\rho }}}}_{{\boldsymbol{PF}}{\boldsymbol{,}}{\bf{\text{MMSE}}}}$	${\hat{{\boldsymbol{\rho }}}}_{{\boldsymbol{i}}{\boldsymbol{,}}{\bf{\text{MMSE}}}}{\boldsymbol{-}}\,{\hat{{\boldsymbol{\rho }}}}_{{\boldsymbol{ATR}}{\boldsymbol{,}}{\bf{\text{MMSE}}}}$	${\hat{{\boldsymbol{\rho }}}}_{{\boldsymbol{i}}{\boldsymbol{,}}{\bf{\text{MMSE}}}}{\boldsymbol{-}}\,{\hat{{\boldsymbol{\rho }}}}_{{\boldsymbol{PF}}{\boldsymbol{,}}{\bf{\text{MMSE}}}}$	${\hat{{\boldsymbol{\rho }}}}_{{\boldsymbol{i}}{\boldsymbol{,}}{\bf{\text{MMSE}}}}{\boldsymbol{-}}\,{\hat{{\boldsymbol{\rho }}}}_{{\boldsymbol{ATR}}{\boldsymbol{,}}{\bf{\text{MMSE}}}}$
i = MDF	0.13 (−0.03, 0.35) 1.51E-01	0.12 (−0.04, 0.34) 2.47E-01	0.15 (−0.16, 0.49) 1.00E + 00	0.03 (−0.29, 0.37) 1.00E + 00	0.12 (−0.08, 0.39) 5.67E-01	0.16 (−0.03, 0.44) 1.21E-01
i = PF	—	−0.01 (−0.20, 0.18) 1.00E + 00	—	−0.11 (−0.45, 0.20) 1.00E + 00	—	0.04 (−0.18, 0.28) 1.00E + 00

Difference between a pair of Pearson correlation coefficients ${\hat{\rho }}_{i,MMSE}$ and ${\hat{\rho }}_{j,MMSE}$ for the total, male and female group, where *i* = {MDF, PF} and j = {PF, ATR}. The rest of the details are identical with Table 3.

### Correlations between the MMSE score, EEG biomarkers and demographic variables

There were correlations between the MMSE score, EEG variables (PF, MDF and ATR) and demographic variables (age and education level), as summarized in Table 2 to Table 5 and pictorially presented in Fig. 2. Table 2 presents PCCs of the MMSE score and each EEG variable with age and education level for the total, male and female participants, and Table 3 presents differences between pairs of PCCs in Table 2. It shows moderate to strong correlations of the MMSE score with age ($-0.61\le {\hat{\rho }}_{{\rm{MMSE}},{\rm{age}}}\le -\,0.40$) and with education level ($0.50\le {\hat{\rho }}_{{\rm{MMSE}},{\rm{edu}}}\le 0.62$). The correlation with age was stronger in females than in males while no such gender difference was observed in the correlation with the educational level (${\hat{\rho }}_{F({\rm{age}})}-{\hat{\rho }}_{M({\rm{age}})}=-\,0.21$; 95% CI: −0.42 to −0.01; p = 0.026). On the other hand, moderate or weak correlations were found between each EEG variable, and age or education level ($-0.43\le {\hat{\rho }}_{i,{\rm{age}}}\le -\,0.19$, where *i* = {MDF, PF, ATR}). For the EEG variables, there was weak or no gender difference in the correlations with age (correlations in females were marginally stronger), while no gender difference was found in the correlation with education level. As shown in Table 3, the correlations of the MMSE score with both age and education level were stronger than that of any EEG variable with age and education level for females, but such differences were reduced or diminished for males. For the interested reader, we offered some representative EEG spectra with the MDF, PF and ATR for decreasing MMSE scores, education level, and advancing age in Fig. S1 in Supplementary Materials.

**Table 6:** Association of EEG variables with the total MMSE and cognitive domain scores.

	MDF			PF			ATR		
	$\hat{{\boldsymbol{\beta }}}$ (95% CI) p-value	${\hat{{\boldsymbol{\beta }}}}^{{\boldsymbol{\ast }}}$ (95% CI)	Adj R^2^	$\hat{{\boldsymbol{\beta }}}$ (95% CI) p-value	${\hat{{\boldsymbol{\beta }}}}^{{\boldsymbol{\ast }}}$ (95% CI)	Adj R^2^	$\hat{{\boldsymbol{\beta }}}$ (95% CI) p-value	${\hat{{\boldsymbol{\beta }}}}^{{\boldsymbol{\ast }}}$ (95% CI)	Adj R^2^
MMSE score (total)	1.05 (0.81, 1.29) 5.04E-17	0.30 (0.23, 0.37)	0.49	0.75 (0.47, 1.02) 1.80E-07	0.19 (0.12, 0.26)	0.44	2.36 (1.43, 3.29) 8.07E-07	0.18 (0.11, 0.25)	0.44
Orientation to time	0.29 (0.23, 0.36) 5.41E-16	0.33 (0.25, 0.40)	0.36	0.21 (0.13, 0.29) 2.81E-07	0.21 (0.13, 0.29)	0.31	0.68 (0.42, 0.95) 7.84E-07	0.20 (0.12, 0.28)	0.31
Orientation to place	0.19 (0.14, 0.24) 7.18E-14	0.31 (0.23, 0.39)	0.30	0.15 (0.09, 0.20) 4.58E-07	0.21 (0.13, 0.29)	0.25	0.42 (0.23, 0.61) 1.72E-05	0.18 (0.10, 0.26)	0.24
Registration	0.05 (0.03, 0.07) 5.73E-05	0.19 (0.10, 0.28)	0.10	0.04 (0.02, 0.07) 1.24E-03	0.15 (0.06, 0.24)	0.08	0.13 (0.04, 0.22) 4.77E-03	0.13 (0.04, 0.22)	0.08
Attention and calculation	0.18 (0.08, 0.28) 5.56E-04	0.14 (0.06, 0.22)	0.31	0.12 (0.00, 0.23) 4.21E-02	0.08 (0.00, 0.16)	0.30	0.30 (−0.08, 0.68) 1.26E-01	0.06 (−0.02, 0.14)	0.30
Recall	0.12 (0.06, 0.18) 2.80E-04	0.16 (0.08, 0.25)	0.15	0.08 (0.01, 0.16) 2.29E-02	0.10 (0.01, 0.19)	0.14	0.30 (0.06, 0.54) 1.56E-02	0.11 (0.02, 0.19)	0.14
Language	0.16 (0.11, 0.20) 2.33E-10	0.28 (0.19, 0.36)	0.22	0.07 (0.02, 0.12) 1.22E-02	0.11 (0.02, 0.20)	0.17	0.33 (0.15, 0.51) 3.88E-04	0.15 (0.07, 0.24)	0.18
Visual construction	0.03 (−0.00, 0.06) 7.48E-02	0.07 (−0.01, 0.15)	0.31	0.03 (−0.00, 0.06) 6.26E-02	0.07 (−0.00, 0.15)	0.31	0.08 (−0.03, 0.18) 1.66E-01	0.06 (−0.02, 0.13)	0.31
Decision-making	0.03 (0.01, 0.06) 2.13E-03	0.14 (0.05, 0.23)	0.11	0.04 (0.02, 0.06) 1.44E-03	0.14 (0.06, 0.23)	0.11	0.13 (0.04, 0.21) 2.61E-03	0.14 (0.05, 0.22)	0.11

Regression coefficients $\hat{\beta }$ corresponding to each EEG variable were obtained from multiple linear regression (MLR) models for each of the MMSE total and sub-domain scores. All MLR models include demographic covariates (sex, age, and education level). The first column ($\hat{\beta }$) of each EEG variable contains the estimated slope, 95% confidence interval for $\hat{\beta }$, and p-values obtained from t-distribution with the 491 degrees of freedom. The second (${\hat{\beta }}^{\ast }$) and third column (Adj ${{\rm{R}}}^{2}$) of each EEG variable indicates standardized regression coefficients and adjusted ${{\rm{R}}}^{2}$, respectively.

Repeatedly, Table 4 presents PCCs between the MMSE score and each EEG variable, and Table 5 presents differences between pairs of PCCs. It shows weak to moderate correlations of the MMSE score with EEG variables ($0.26\le {\hat{\rho }}_{i,{\rm{MMSE}}}\le 0.52$, where *i* = {MDF, PF, ATR}), and no difference was found between genders. As shown in Table 5, the MDF showed marginally stronger correlation with the MMSE score than PF or ATR. In summary, Tables 2 to 5 indicate that the MMSE score and EEG variables decreased with advancing age and less-educated participants, and the tendency was stronger for females.

**Table 7:** Model evaluation results of the predictive models.

Model	*λ*	RMSE* (10-CV)	RMSE# (test)	${\hat{{\boldsymbol{\rho }}}}_{({{\boldsymbol{y}}}_{{\bf{t}}{\bf{e}}{\bf{s}}{\bf{t}}},{\hat{{\boldsymbol{y}}}}_{{\bf{t}}{\bf{e}}{\bf{s}}{\bf{t}}})}$¶ (95% CI)	${\hat{{\boldsymbol{\delta }}}}_{{\bf{t}}{\bf{e}}{\bf{s}}{\bf{t}}}$†	ICC‡ (95% CI)
WLS	NA	3.423	2.767	0.746 (0.644, 0.822)	0.355 ± 2.758	0.746 (0.647, 0.823)
Ridge (*α* = 0.0)	0.003	3.421	2.699	0.756 (0.657, 0.829)	0.282 ± 2.698	0.756 (0.655, 0.833)
Elastic net (*α* = 0.9)	0.007	3.410	2.687	0.757 (0.659, 0.830)	0.319 ± 2.681	0.757 (0.665, 0.827)
LASSO (*α* = 1.0)	0.007	3.408	2.685	0.758 (0.659, 0.830)	0.327 ± 2.678	0.757 (0.660, 0.828)

The penalized models shown in the table are the best models selected from all possible models (a total of 3300) generated with *λ* (exp[log(10)] to (exp[log(0.0001)], a total of 300) and α (0.0 to 1.0, a total of 11) grids, where λ is the regularization parameter for the penalized regression (selected from the 10-fold cross-validation) and α is the mixing parameter for the elastic net (ridge: α = 0; LASSO: α = 1).

^*^Root mean squared error (RMSE) resulting from the 10-fold cross-validation based on the training set.

^¶^Pearson correlation coefficients ($\hat{{\boldsymbol{\rho }}})$ and their 95% confidence intervals (95% CI).

^†^Mean difference (${\hat{{\boldsymbol{\delta }}}}_{{\bf{t}}{\bf{e}}{\bf{s}}{\bf{t}}}$) between the true and predicted MMSE scores and their standard deviations.

^‡^Intra-class correlation coefficients (ICC) and their 95% confidence intervals estimated through 1000 bootstrap samples.

The 10-fold validation results for penalized regression models are presented in Fig. S3 in Supplementary Materials.

### Association of EEG biomarkers with MMSE cognitive domains

To investigate the association between the resting-state EEG variables and the MMSE domain scores, we performed unadjusted and partial Pearson correlation tests (Fig. 3(A)). The unadjusted PCC results showed that all MDF, PF and ATR variables were weak to moderately correlated with the total MMSE scores and the 8 cognitive domain scores: ${\hat{\rho }}_{{\rm{MDF}},{\rm{OT}}}=0.474$ (95% CI: 0.359 to 0.576; p = 9.08E-28, highest correlation) and ${\hat{\rho }}_{{\rm{ATR}},{\rm{RG}}}=0.199$ (95% CI: 0.061 to 0.329; p = 2.15E-4, lowest correlation), where OT and RG are abbreviations of “orientation to time” and “registration”, respectively. As expected from the above results, the partial PCC, controlled for sex, age and education level, was reduced with lowered confidence intervals. Specifically, the MDF remained correlated with most of the MMSE cognitive domains, except for the domains of visual construction and decision making. In the case of the PF and ATR, correlations became insignificant with additional domains such as attention and calculation, and recall. The highest partial PCC and significance level was observed between the MDF and orientation to time: ${\hat{\rho }}_{{\rm{MDF}},{\rm{OT}}}=0.354$ (95% CI: 0.226, 0.470; p = 1.19E-14).

To test the relative strength of correlation between cognitive domains and each EEG variable, in Fig. 3(B), we presented the difference of pairs of unadjusted and partial PCCs between the OT domain ${\hat{\rho }}_{i,OT}$ and other domains (${\hat{\rho }}_{i,j}$), with *i* = {MDF, PF, ATR} and j={domain indices other than OT}. It shows that, for all of the MDF, PF and ATR, the correlation with orientation to time and place were equally strong in terms of the unadjusted PCC and partial PCC which was adjusted for sex, age and education level.

The results of the multiple linear regression analyses performed to examine the association between EEG variables and the total MMSE and domain scores are shown in Table 6. The regression model used sex, age and education level as common covariates of each EEG variable (MDF, PF and ATR). Positive associations ($\hat{\beta }$) were obtained in all regression models for the three EEG variables. The standardized regression coefficient (${\hat{\beta }}^{\ast }$) indicate the relative contribution of the associated variable to predict the total MMSE and cognitive domain scores; each EEG variable was moderately associated with the total MMSE, orientation to time and orientation to place scores (0.30, 0.33 and 0.31, respectively, for the MDF; 0.19, 0.21 and 0.21 for the PF; and 0.18, 0.20 and 0.18 for the ATR). Consistently, the adjusted R^2^ of each regression model moderately explained the variances in the total MMSE (R^2^ = 0.49 for MDF, 0.44 for both PF and ATR), orientation to time (R^2^ = 0.36 for MDF, 0.31 for both PF and ATR) and orientation to place scores (R^2^ = 0.30 for MDF, 0.25 for PF and 0.24 for ATR).

### Predictive models for the MMSE using the EEG and demographic variables

#### Predictive model for the MMSE total score

Finally, we developed predictive models for the MMSE using the EEG variables to examine the possibility of replacing or supplementing the MMSE with resting EEG slowing combined with demographic information. As details were described in Section 2.5, we adopted a double cross-validation procedure for a realistic estimation of prediction errors of each model.

**Figure 4:**
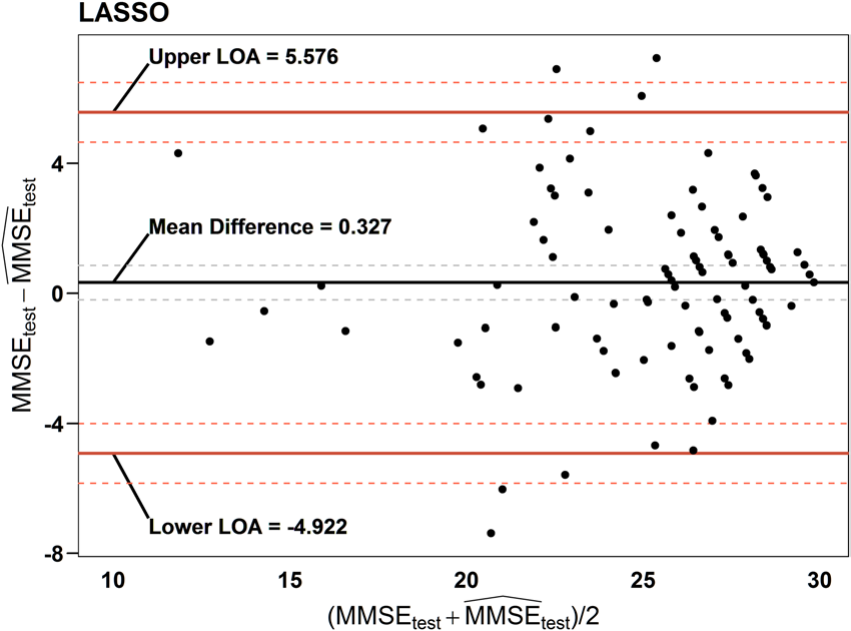
Bland-Altman plot for the final predictive model (LASSO). Bland-Altman plot of the agreement between the true MMSE (***MMSE***_***test***_) and predicted MMSE scores (${\widehat{{\boldsymbol{MMSE}}}}_{{\boldsymbol{test}}}$) for the test set based on the LASSO. Dashed lines between upper and lower value of statistics represented by solid lines indicate the approximate 95% confidence intervals for mean difference, upper and lower limits of agreement (LOA), respectively.

Table 7 shows the best prediction results of the four approaches (the 10-fold validation results for penalized regression models are presented in Fig. S3 in Supplementary Materials). As a result, all four regression models (WLS, LASSO, elastic net and ridge) predicted the true MMSE score with the RMSE ranging between 2.767 (WLS) and 2.685 (LASSO). Relatedly, the associated PCCs (${\hat{\rho }}_{({y}_{{\rm{test}}},{\hat{y}}_{{\rm{test}}})}$) were between 0.746 (WLS) and 0.758 (LASSO), with 95% confidence intervals between 0.644 and 0.830, and ICCs were between 0.746 (WLS) and 0.757 (Elastic net, LASSO), and with 95% confidence intervals between 0.647 and 0.828. These results imply moderate to good agreement of the regression models. The finally chosen model was LASSO with the minimum RMSE of 2.685. The detailed model equation was described in Table 8 and a Bland-Altman plot for the final model was employed to investigate the LOA between the true and predicted MMSE values in Fig. 4; a lower LOA of −4.922 and an upper LOA of 5.576 was obtained. The two equations in Table 8 were derived from a single equation (1 for female and 0 for male). For both the male and female equations, the MDF was the primary predictor.

**Table 8:** The final model (LASSO).

Predicted MMSE	Equation
${\widehat{{\rm{MMSE}}}}_{{\rm{Male}}}=$	24.597 + 7.216 MDF − 6.318 MDF^2^ − 0.013 PF + 0.913 ATR − 0.93 ATR^2^ + 4.192 Age − 5.06 Age^2^ + 4.725 Edu − 2.523 Edu^2^
${\widehat{{\rm{MMSE}}}}_{{\rm{Female}}}=$	24.597 + 7.216 MDF − 6.343 MDF^2^ + 0.008 PF + 0.4 PF^2^ + 1.525 ATR − 0.939 ATR^2^ + 4.192 Age − 5.502 Age^2^ + 3.277 Edu − 1.476 Edu^2^

Equations are obtained from the LASSO with λ = 0.007 (α = 1).

## Discussion

The present results complemented previous evidence showing that the alpha rhythms of resting-state EEG constitute an important neural substrate for human cognition^60–63^. The significant enhancement of scalp alpha rhythms in the resting condition is usually considered a sign of wakeful brain idling and inhibitory processes^63^. These alpha rhythms are mainly modulated by thalamo-cortical and cortico-cortical interactions^64^. Previous studies have shown that slowing and attenuation of resting alpha rhythms are correlated with declines in attention and memory function^65–68^. In terms of band powers, diverse behaviors were reported. Subjects with good memory performance were reported to have significantly larger upper alpha, but less theta and lower alpha power, and good calculation performers have more beta and theta power than bad performers^69^. On the other hand, Doppelmayr *et al*. reported a positive correlation between intelligence and alpha power; upper alpha was related to the ability to process semantic information, whereas the two lower alpha bands were associated with attentional demands that dominate during the encoding of new information^70^. High-power alpha rhythms predict good cognitive performance, specifically, completing a specific function or task with few errors and high efficiency^71,72^. The interpretation needs more caution with event-related paradigms with different levels of task difficulties^71,73^. To test the hypothesis that alpha rhythms are related to cognitive performance, some studies have attempted to enhance the relative power of alpha rhythms by alpha-neurofeedback training programs. These studies found that cognitive performance was improved only in subjects who showed high-power alpha rhythms^74,75^.

In this study, we reported moderate correlations between resting EEG biomarkers and neuropsychological MMSE scores and examined the possibility that resting EEG biomarkers could replace or supplement the MMSE scores. Resting EEG biomarkers reflect the degeneration of the dominant oscillatory frequencies of the alpha or alpha-like types, and the MMSE evaluates global cognitive status in aged populations. The results of our analysis of 496 local community residents who were 50 years of age or older suggest that the MDF is the best biomarker for MMSE cognitive scores among the three tested EEG biomarkers (MDF, PF and ATR). Regarding the tertile split categorization of MMSE cognitive stages (T3 (MMSE 28–30), T2 (MMSE 25–27) and T1 (MMSE ≤ 24)), resting-state EEG slowing was significant between the cognitive decline stages of T2 → T1 for both sexes (Fig. 2). The MMSE is primarily used as a screening tool for dementia, with scores below 24 commonly used to indicate a cognitive deficit, and this test has a ceiling effect in young healthy adults^76^. Therefore, resting-state EEG slowing across the T2 → T1 stages was considered an appropriate complementary or alternative tool for the MMSE, particularly in the early or moderate stages of cognitive decline.

The MMSE is brief and simple and is a popular screening tool used to assess cognitive impairment worldwide. The MMSE is influenced by non-explicit cognitive variables, such as age and education level^77^, as observed in our study (Tables 2 and 3). There are still debates as to whether the cutoff value of the MMSE score should be different for different ages and education levels, and if so, to what degree^77^. Similarly to the MMSE score, the resting-state EEG results were found to correlate with age and education level (Tables 2 and 3). However, the physiological mechanism underlying the association of the EEG results with age and education level are different from that of the MMSE results with these two factors; The MMSE tests the cognition but resting-state EEG measurements reflect background brain electrical activity in the awake idling state^78^, In answering to any cognitive questionnaire such as the MMSE, complex cognitive processes are involved, such as perception, recognition, comprehension, memorizing, reasoning, planning, language usage, and problem solving^79^. For a more explicit comparison with the MMSE, a further study with additional EEG paradigms of event-related potential related to perception, attention, or memory recall would be useful^13,80^.

In examining the association between the EEG biomarkers and each MMSE cognitive domain, namely, orientation to time and place, memory registration and recall, attention and calculation, language, visual construction, and decision making, we found the strongest association between EEG variables (either MDF, PF or ATR) and the orientation to time and place, and the level of association was affected by sex, age or education level (Fig. 3 and Table 6). Previously, cognitive impairment in memory recall was first reported to occur first among community-dwelling AD patients, followed by impairments in the orientation to time, attention and concentration, orientation to place, language, visual construction, and memory registration MMSE domain scores^81^. Therefore, our findings suggest that resting-state EEG slowing is most susceptible to the loss of orientation to time and place relative to other cognitive domains, which did not coincide with the progression of impairment in cognitive domains among AD patients.

Finally, we developed the linear prediction models (WLS, ridge, elastic net and LASSO) for the MMSE using the resting EEG biomarkers and cognition-dependent demographic information such as sex, age and education level (Table 7), and obtained the RMSEs of 2.767~2.685, PCCs of 0.746~0.758 and ICCs of 0.746~757. It implies that the model-dependent effect was minor in the correlation between the EEG biomarkers and the MMSE score. In the selected regression model (Table 7, LASSO), the MDF was the leading predictor, and age and education level ranked second and third. These results indicate that the resting-state EEG biomarkers of MDF, PF and ATR have the potential to replace or supplement the MMSE for cognitive status evaluation. Through the test in classification performance between T1 group and (T2,T3)-combined group in the MMSE cognitive stages (Tables S2 and S3 and Fig. S4 in Supplementary Materials), the EEG biomarkers showed moderate level of AUC value (0.849) and accuracy (0.754), which again confirmed the possibility of the resting-state EEG biomarkers as a substitute or supplement of the MMSE^82^.

Recently, it was suggested that the entropy markers (or complexity) of EEG were correlated with the MMSE scores of 79 probable AD subjects. Specifically, some studies reported that reductions in complexity, by means of auto-mutual information, spectral entropy, and multiscale entropy, were observed as the MMSE scores decreased^83^. These findings prompt further investigations into correlations with EEG biomarkers in our study cohort.

### Limitation

This study has some limitations. First, we compared EEG biomarkers with only MMSE scores. The MMSE offers modest accuracy for the screening of dementia, but its specificity and sensitivity in mild cognitive impairment (MCI) are known to be poor^82^. Since the subjects in this study were mainly in the range of cognitive normal – MCI – mild dementia, more sensitive tools for assessing early cognitive declines, such as the Addenbrooke’s Cognitive Examination^84^ or the CERAD instrument^85^, would have been beneficial. Second, it is a cross-sectional study; a follow-up study with changes in scores of a neuropsychological test in 1~2 years after the initial assessment is needed to clinically validate the three EEG variables.

## Conclusion

In conclusion, by comparing the neuropsychological MMSE scores and resting EEG slowing measured in the prefrontal regions of 496 elderly participants, we observed a moderate correlation between resting EEG slowing and the MMSE global and cognitive domain scores, especially for orientation to time and place. We developed a predictive model for the MMSE scores using three resting EEG biomarkers and demographic covariates with a Pearson correlation coefficient of 0.758. The annual progression rate of MCI to dementia is reported to be 10–15%^86^, which requires frequent cognitive status assessments to ensure the appropriate clinical care of MCI patients. In a future work, the development of resting-state EEG slowing into a screening tool could be attempted that can replace the MMSE particularly for assessing the early stages of cognitive decline. A predictive model of cognitive impairment could be developed, e.g., based on a longitudinal study, by investigating associations with a comprehensive neuropsychological battery such as the CERAD neuropsychological instrument^87^ or with MRI or PET imaging.

## Supplementary information

Supplementary materials
