# Supplementary material for: Resting-state prefrontal EEG biomarkers in correlation with MMSE scores in elderly individuals
Source: Sci Rep. 2019 Jul 18;9:10468. doi: 10.1038/s41598-019-46789-2 (PMC6639387; doi:10.1038/s41598-019-46789-2)
Supplement: Supplementary file 1 — Supplementary materials [file 41598_2019_46789_MOESM1_ESM.docx]

Resting-state prefrontal EEG biomarkers in correlation with MMSE scores in elderly individuals

Supplementary Materials

Jungmi Choi^[[1]](#footnote-1)^, Boncho Ku^[[2]](#footnote-2)^, Young Gooun You^[[3]](#footnote-3)^, Miok Jo^3^, Minji Kwon^3^, Youyoung Choi^3^, Segyeong Jung^3^, Soyoung Ryu^3^, Eunjeong Park^3^, Hoyeon Go^[[4]](#footnote-4)^, Gahye Kim^2^, Wonseok Cha^1^, and Jaeuk U. Kim^2,*^

**Table S1**: Results of the mean differences, Pearson correlation coefficients and intraclass correlation coefficients (ICC) for resting-state EEG slowing measured between the prefrontal regions (Fp1 and Fp2) and occipital regions (O1 and O2).

| **Region** | **EEG variables** | **Mean difference (95% CI)** | **Pearson** $\boldsymbol{\rho}$ **(95% CI)** | **ICC (95% CI)** |
| --- | --- | --- | --- | --- |
| Fp1 vs. O1 | MDF | 0.078   (-0.376, 0.532) | 0.875   (0.755, 0.938) | 0.865   (0.730, 0.932) |
|  | PF | 0.035   (-0.292, 0.361) | 0.937   (0.872, 0.969) | 0.936   (0.873, 0.968) |
|  | ATR | 0.086   (-0.457, 0.630) | 0.572   (0.273, 0.770) | 0.541   (0.241, 0.738) |
| Fp2 vs. O2 | MDF | 0.012   (-0.766, 0.790) | 0.692   (0.447, 0.840) | 0.690   (0.432, 0.832) |
|  | PF | 0.060   (-0.210, 0.331) | 0.958   (0.914, 0.980) | 0.951   (0.896, 0.977) |
|  | ATR | 0.091   (-0.636, 0.818) | 0.503   (0.180, 0.727) | 0.440   (0.114, 0.669) |


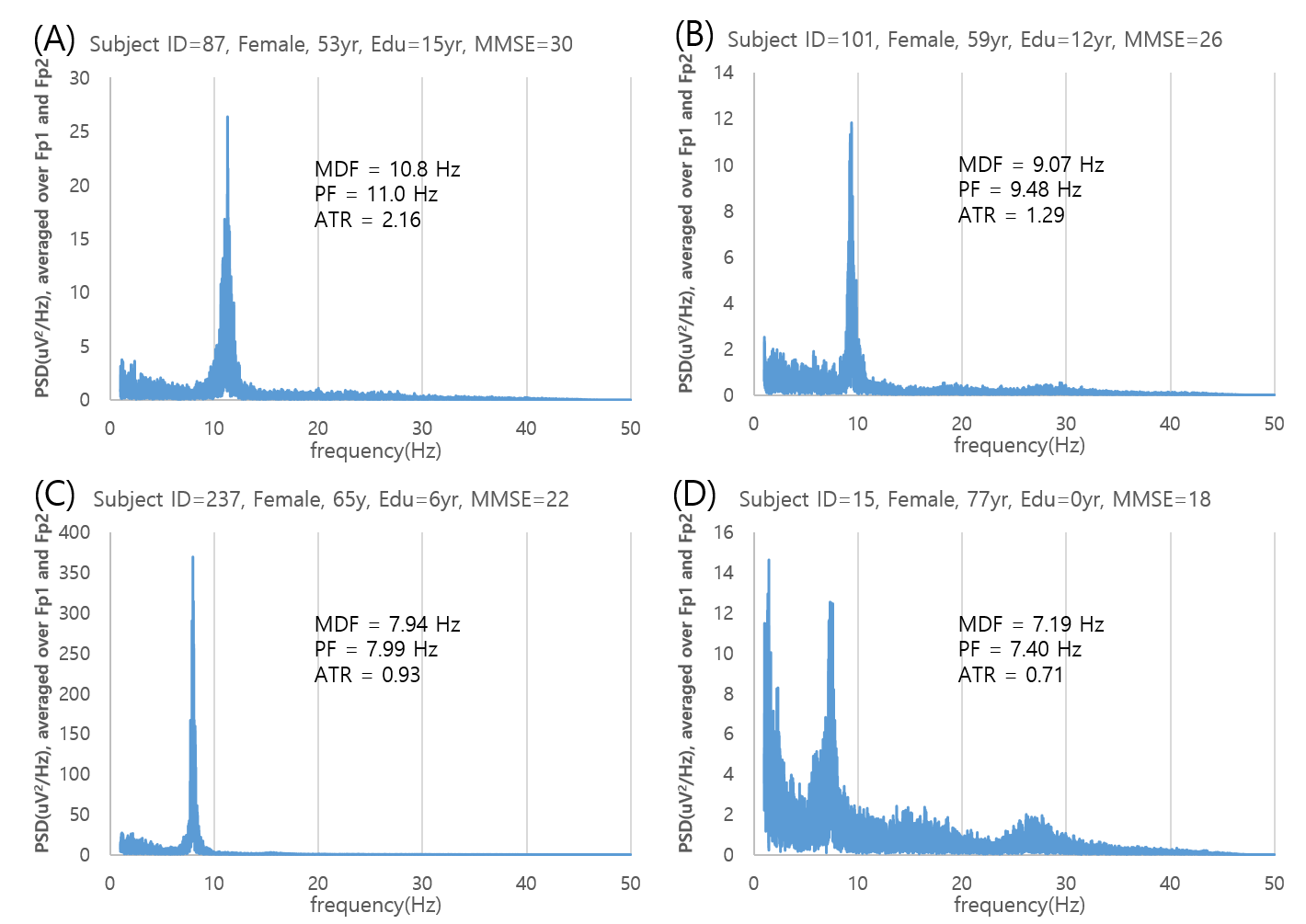


**Figure S1**: Representative EEG spectra with the MDF, PF and ATR for decreasing MMSE score, education level, and advancing age from (A) to (D). Female participant with (A) MMSE=30, education level = 15 years and 53 years old, (B) MMSE=26, education level = 12 years and 59 years old, (C) MMSE=22, education level = 6 years and 65 years old, (D) MMSE=18, education level = 0 years and 77 years old.


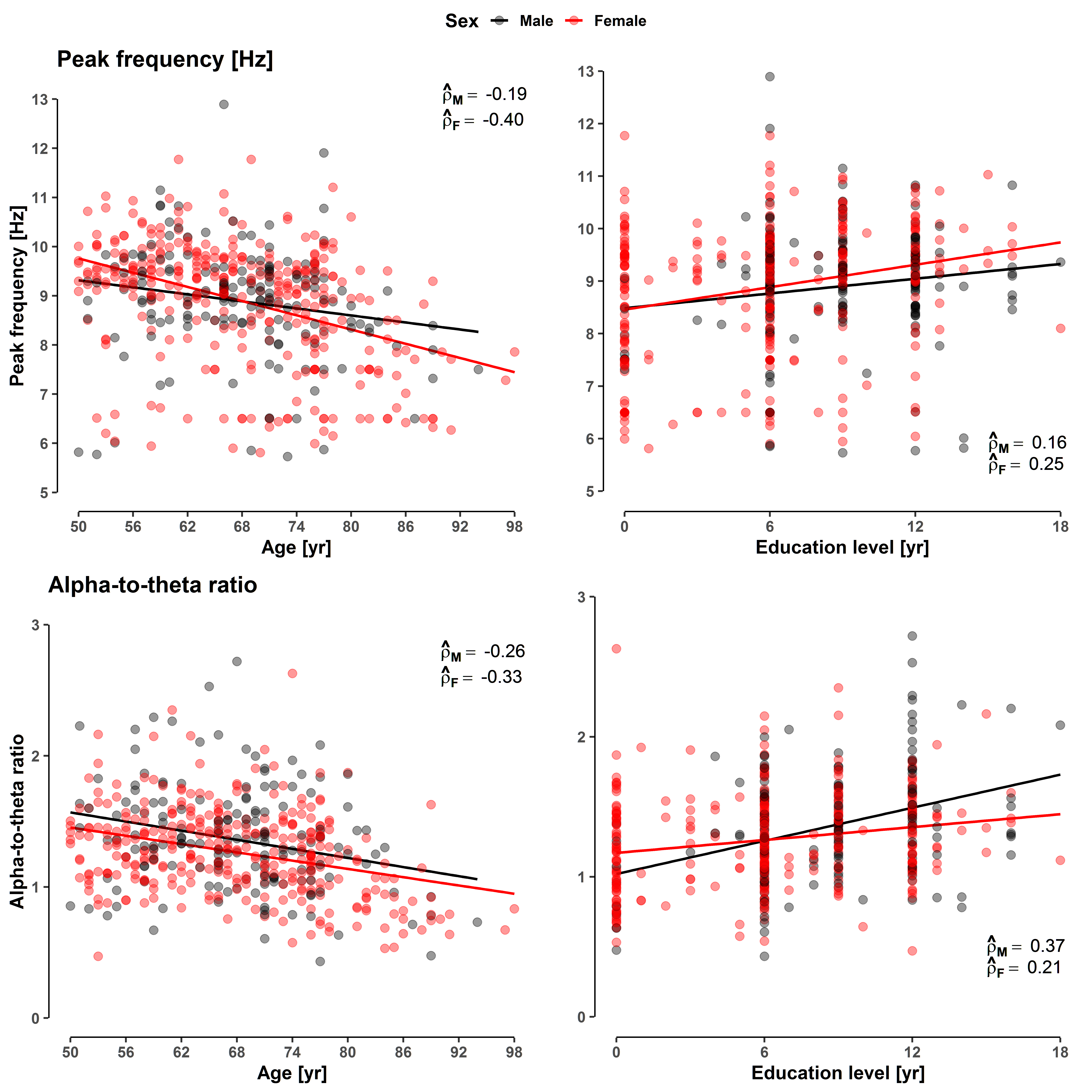


**Figure S2**: Scatterplots between the EEG variables (PF and ATR) and demographic variables (age and education levels) according to sex (supplement of Fig. 2). The simple linear regression curves for the MMSE and each EEG variable are plotted according to sex. The Pearson correlation coefficients between demographic variables and EEG variables are noted on each panel.


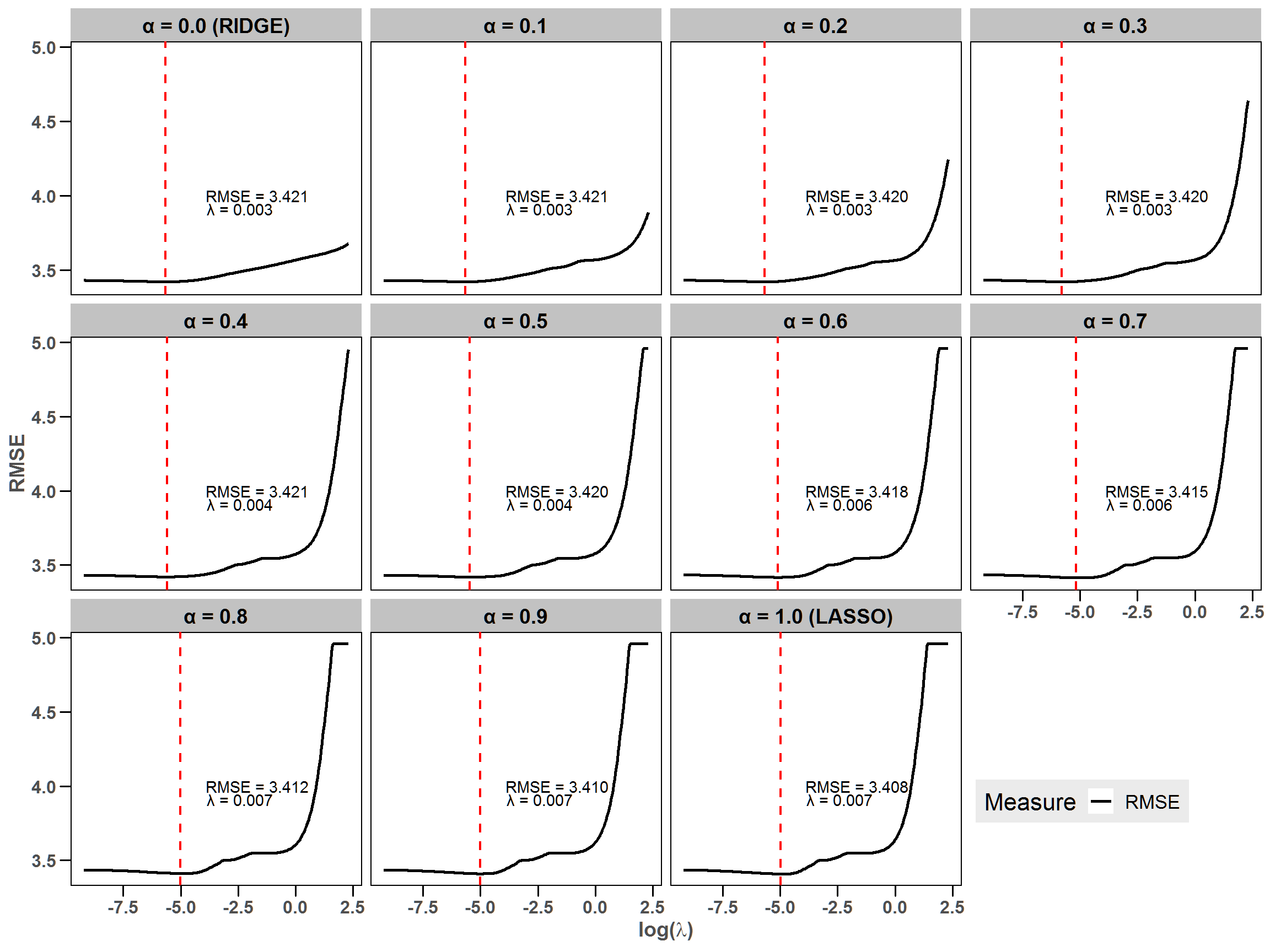


**Figure S3**: 10-fold cross-validation results for penalized regression models

### Building predictive models for the MMSE tertiles (T1 vs. T2 ~ T3)


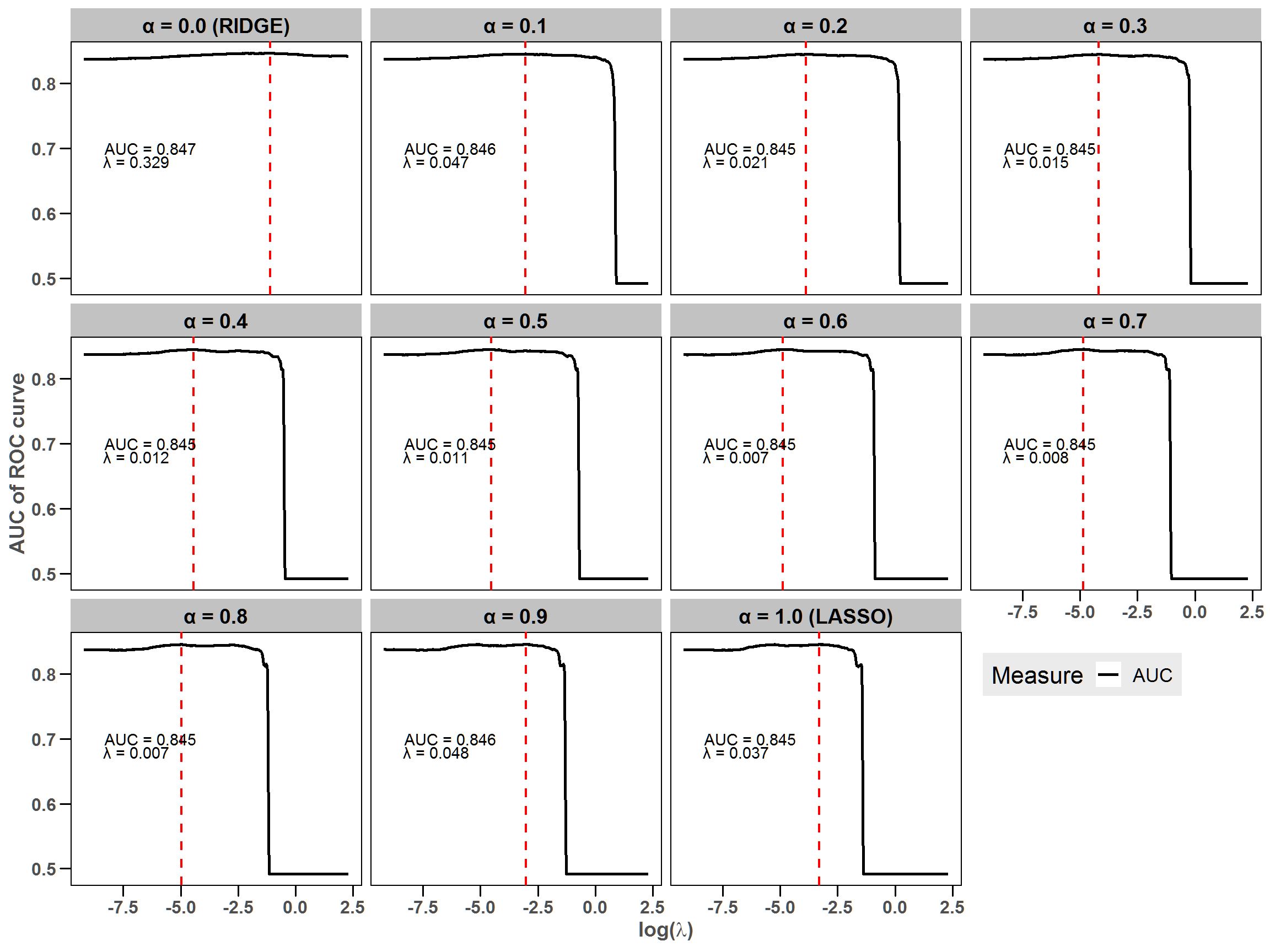


**Figure S4**: 10-fold cross-validation results for penalized logistic regression models (stratified by T1 and T2~T3)

#### Table S2: Validation results (selected model according to the model frameworks: stratified by T1 and T2~T3)

|  | | **10-fold CV** | |  | **Validation (test)** | |
| --- | --- | --- | --- | --- | --- | --- |
| **Model** | **\lambda** | **AUC** | **Deviance** |  | **AUC** | **Deviance** |
| WLS | NA | 0.824 | 361.1 |  | 0.807 | 98.0 |
| Ridge (\alpha = 0.0) | 0.329 | 0.847 | 356.2 |  | 0.844 | 92.6 |
| Elastic net (\alpha = 0.2) | 0.048 | 0.846 | 352.2 |  | 0.849 | 90.6 |
| LASSO (\alpha = 1.0) | 0.037 | 0.845 | 348.7 |  | 0.848 | 89.7 |
| The penalized models shown in the table is the best model selected from all possible models (a total of 5500) generated with $\lambda$ ($exp[log(10)]$ to $exp [log(0.0001)],$ a total of 300) and $\alpha$ (0.0 to 1.0, a total of 11) grids.   \lambda: tuning parameters for the penalized logistic regression (selected based on 10-fold cross-validation); \alpha: mixing parameters for the elastic net (ridge: $\alpha= 0$; LASSO: $\alpha= 1$). | | | | | | |

#### Table S3: Performance of the selected models after adopting optimal thresholds (stratified by T1 and T2~T3)

|  | |  | **10- fold CV** | | | |  | **Validation (test)** | | | | |
| --- | --- | --- | --- | --- | --- | --- | --- | --- | --- | --- | --- | --- |
| **Model** | **Threshold** |  | **Se** | **Sp** | **Prc** | **Acc** |  | **Se** | **Sp** | **Prc** | **Acc** |  |
| WLS | 0.271 |  | 0.758 | 0.765 | 0.595 | 0.763 |  | 0.710 | 0.725 | 0.537 | 0.720 |  |
| Ridge | 0.321 |  | 0.750 | 0.754 | 0.581 | 0.753 |  | 0.774 | 0.768 | 0.600 | 0.770 |  |
| Elastic net | 0.313 |  | 0.758 | 0.765 | 0.595 | 0.763 |  | 0.742 | 0.739 | 0.561 | 0.740 |  |
| LASSO | 0.310 |  | 0.758 | 0.765 | 0.595 | 0.763 |  | 0.742 | 0.754 | 0.575 | 0.750 |  |
| Thresholds were determined based on the method introduced by Riddle et al^1^, which maximizes sensitivity and specificity simultaneously. All thresholds were obtained from the training results of the selected model.  Se: sensitivity; Sp: specificity; Prc: precision; Acc: accuracy | | | | | | | | | | | | |

### References

1. Riddle, Daniel L., and Paul W. Stratford. "Interpreting validity indexes for diagnostic tests: an illustration using the Berg balance test." *Physical therapy* 79.10 (1999): 939-948.

1. Human Anti-Aging Standards Research Institute, Uiryeong-gun, Gyeongsangnam-do, Republic of Korea [↑](#footnote-ref-1)
2. Korea Institute of Oriental Medicine, Yusung-gu, Deajon, Republic of Korea [↑](#footnote-ref-2)
3. Uiryeong Community Health Center, Uiryeong-gun, Gyeongsangnam-do, Republic of Korea [↑](#footnote-ref-3)
4. Semyung University, Jecheon-si, Chungcheongbuk-do, Republic of Korea

   * Correspondence to jaeukkim@kiom.re.kr [↑](#footnote-ref-4)
